# Disruption of Basal Lamina Components in Neuromotor Synapses of Children with Spastic Quadriplegic Cerebral Palsy

**DOI:** 10.1371/journal.pone.0070288

**Published:** 2013-08-16

**Authors:** Karyn G. Robinson, Janet L. Mendonca, Jaimee L. Militar, Mary C. Theroux, Kirk W. Dabney, Suken A. Shah, Freeman Miller, Robert E. Akins

**Affiliations:** 1 Nemours Biomedical Research, Alfred I. duPont Hospital for Children, Wilmington, Delaware, United States of America; 2 Department of Anesthesiology and Critical Care, Alfred I. duPont Hospital for Children, Wilmington, Delaware, United States of America; 3 Department of Orthopedic Surgery, Alfred I. duPont Hospital for Children, Wilmington, Delaware, United States of America; Virginia Tech Carilion Research Institute, United States of America

## Abstract

Cerebral palsy (CP) is a static encephalopathy occurring when a lesion to the developing brain results in disordered movement and posture. Patients present with sometimes overlapping spastic, athetoid/dyskinetic, and ataxic symptoms. Spastic CP, which is characterized by stiff muscles, weakness, and poor motor control, accounts for ∼80% of cases. The detailed mechanisms leading to disordered movement in spastic CP are not completely understood, but clinical experience and recent studies suggest involvement of peripheral motor synapses. For example, it is recognized that CP patients have altered sensitivities to drugs that target neuromuscular junctions (NMJs), and protein localization studies suggest that NMJ microanatomy is disrupted in CP. Since CP originates during maturation, we hypothesized that NMJ disruption in spastic CP is associated with retention of an immature neuromotor phenotype later in life. Scoliosis patients with spastic CP or idiopathic disease were enrolled in a prospective, partially-blinded study to evaluate NMJ organization and neuromotor maturation. The localization of synaptic acetylcholine esterase (AChE) relative to postsynaptic acetylcholine receptor (AChR), synaptic laminin β2, and presynaptic vesicle protein 2 (SV2) appeared mismatched in the CP samples; whereas, no significant disruption was found between AChR and SV2. These data suggest that pre- and postsynaptic NMJ components in CP children were appropriately distributed even though AChE and laminin β2 within the synaptic basal lamina appeared disrupted. Follow up electron microscopy indicated that NMJs from CP patients appeared generally mature and similar to controls with some differences present, including deeper postsynaptic folds and reduced presynaptic mitochondria. Analysis of maturational markers, including myosin, syntrophin, myogenin, and AChR subunit expression, and telomere lengths, all indicated similar levels of motor maturation in the two groups. Thus, NMJ disruption in CP was found to principally involve components of the synaptic basal lamina and subtle ultra-structural modifications but appeared unrelated to neuromotor maturational status.

## Introduction

Cerebral palsy (CP), which is one of the most common causes of physical disability in children, is a group of movement disorders occurring when a static encephalopathy develops in the fetal or infant brain [1], [2]. Patients with CP have difficulty with movement, coordination, and balance associated with weakness, poor muscle control, and spasticity [3], [4]. Significant research has focused on the genesis and prevention of central nervous system (CNS) injury in CP; however, the mechanisms and manifestations of peripheral motor dysfunction are only partially understood [1], [5].

Clinically, CP patients are classified as spastic (stiff muscles), athetoid (writhing movements), or ataxic (poor balance and coordination). Spastic CP accounts for about 80% of all cases. Patients with spastic CP often require lifelong support and endure multiple surgical procedures to correct musculoskeletal problems associated with their condition. Unfortunately, therapeutic approaches are limited because the cellular and molecular mechanisms that contribute to the neuromotor and musculoskeletal manifestations of spastic CP are not completely characterized, although there are clear indications that the peripheral neuromotor system is disrupted in CP patients.

In surgical settings, spastic CP patients exhibit altered sensitivities to neuromuscular blocking agents [6], [7] that are specific for the postsynaptic acetylcholine receptors (AChRs) expressed by muscle at neuromuscular junctions (NMJs). CP patients tend to be more sensitive to the depolarizing agent succinylcholine [7] and more resistant to the non-depolarizing agent vecuronium [6]. These altered sensitivities suggest that NMJs, which link the nervous and musculoskeletal systems, are disrupted in CP patients. Gene expression studies have established that spastic CP patients have distinct expression profiles [8], and cross-sectional studies of CP patients have demonstrated that a subset of NMJs appear disorganized such that AChR, which is present on the post synaptic muscle membrane, is found outside the distribution of synaptic acetylcholinesterase (AChE), which defines the functional limits of the NMJ. Matched controls, on the other hand, exhibited nearly complete overlap of the two staining patterns [9]. Both the average degree of AChR to AChE mismatch and the proportion of highly disorganized NMJs (*i.e.* those with mismatches greater than two standard deviations above control values) present in leg muscle biopsies correlated with the degree of gross motor disability in CP patients [10]. Although these previous microanatomic studies established that dysmorphic NMJs were present, they did not distinguish whether AChE, AChR, or both were maldistributed in CP, nor did they establish whether other critical NMJ components were maldistributed relative to either AChR or AChE.

The presence of morphologically disrupted NMJs in children, adolescents, or adults is unanticipated [11]. The formation of tightly apposed pre- and postsynaptic specializations at NMJs are hallmarks of neuromotor maturation, a process that is generally completed during infancy [11], [12]. Non-colocalization of NMJ components is generally indicative of hereditary disease [13], nerve degeneration/regeneration [14], or incomplete neuromotor maturation [11]. Since the CNS injury in CP occurs during neuromotor maturation, we hypothesized that synaptic disruption in CP patients was associated with the prolongation of neuromotor immaturity.

Accordingly, we investigated the extent of NMJ disorganization in CP by comparing muscle acquired from scoliotic patients undergoing spinal fusion surgery and carrying a diagnosis of either spastic CP or idiopathic scoliosis (IS). Comparison of patients with these two conditions allowed us to access a significant number of surgical cases in which the same anatomic location was exposed during surgery and allowed the potential effects of scoliosis to be segregated from any effects of a CP diagnosis since both groups were scoliotic. The relative distributions of presynaptic (synaptic vesicle protein 2/SV2), synaptic (AChE and laminin β2), and postsynaptic (AChR) markers were quantified by fluorescence microscopy to assess NMJ organization. Ultrastructural differences were evaluated using transmission electron microscopy. The relative maturational status of the muscle samples was determined based on telomere length estimation and on the expression of myosin heavy chain (MYH) isoforms, AChR subunits, myogenin, and syntrophins, which are markers that exhibit differential expression during motor maturation [11], [13], [15], [16], [17], [18], [19], [20].

## Methods

### Patient enrollment and sample acquisition

Patients were enrolled in two distinct phases of research. In the first phase, samples were analyzed using light microscopy and bio-molecular assays and in the second phase, samples were examined using transmission electron microscopy. In both phases, prospective, non-randomized, and partially blinded designs were employed. Patients were enrolled after IRB approval of the study and informed consents and assents were obtained. Patients with a history of botulinum toxin treatment within the 12 months preceding surgery and any patients carrying a diagnosis of chromosomal disorder, degenerative neurological disease, muscular dystrophy, or a diagnosis of neuromuscular scoliosis other than CP were excluded. A complete list of exclusionary criteria and a table summarizing the ages, genders, physical characteristics, and degree of scoliosis are included in detailed methods in Text S1, and Table S1 in Text S1. Muscle biopsies (approximately 1 cm^3^) were obtained during posterior spinal fusion surgery from the lateral aspect of the *spinalis* at the thoraco-lumbar junction on the concave side of the curve from patients with spastic quadriplegic/quadriparetic CP (n = 34 for phase one and n = 5 for phase two) or IS (n = 36, phase one; n = 5 phase two). This combination of patients was selected to minimize potential effects due to scoliosis and to allow for the collection of tissue from the same anatomic location in both patient groups.

Samples from patients enrolled in phase one received a blinding code and were immediately snap-frozen in liquid nitrogen-chilled isopentane and stored at −80°C. Among the samples collected, 7 CP and 6 IS were subsequently excluded from the study due to a lack of motor endplates on microscopic examination. When possible, all light microscopic and biomolecular assays were performed on each biopsy, however, since biopsy samples were necessarily of a limited size, it was not possible to perform all phase-one analyses on each biopsy. Specific sample usage is indicated in the text.

Samples from patients enrolled in phase two of the study were placed into sterile specimen containers immersed in wet ice, and within 10 minutes of excision, placed in freshly prepared 4% paraformaldehyde (PFA; EMS, Hatfield, PA) in 0.1 M Sorensen's phosphate buffer (PB,EMS, Hatfield, PA) at pH 7.4. The tissue was then cut into 1×2 mm pieces and placed in fresh fixative (4% PFA in PB) for further processing as described below.

For some assays, additional control samples were obtained from research tissue banks. De-identified muscle from three patients with Duchenne muscular dystrophy (DMD) were obtained from the Cooperative Human Tissue Network (CHTN) at Nationwide Children's Hospital, Columbus, OH and the NICHD Brain and Tissue Bank for Developmental Disorders at the University of Maryland, Baltimore, MD (these were labeled as thigh muscle, upper arm muscle, and psoas). De-identified muscle from a normal pediatric patient (lower leg) and a pre-term donor (86 days gestation) were obtained from the CHTN at the Ohio State University and the University of Washington Birth Defects Research Laboratory, respectively.

### Fluorescence staining and calculation of appositional scores

Slides containing 8 µm thick cryosections of samples from all patients enrolled in phase one were analyzed. Samples in which fewer than 15 different synapses were found in the sections analyzed were excluded from the analysis. Samples (n = 25 patients with CP, 28 control patients with IS) were triple stained with tetramethylrhodamine-conjugated α-bungarotoxin (Btx; Molecular Probes), biotinylated AE-2 antibody, and hybridoma supernatant containing a monoclonal antibody to either laminin β2 (C4; Developmental Studies Hybridoma Bank [DSHB]) or SV2 (DSHB) (see detailed methods in Text S1).

Samples were digitally imaged on an Olympus BX-60 fluorescence microscope equipped with an Evolution QEi monochrome 12-bit (1360×1036 pixels) digital camera (Media Cybernetics) controlled by Image Pro Plus software (version 5.1; Media Cybernetics). For each fluorophore staining pattern, a threshold value was determined based on the number of pixels at each gray level in the acquired image. The majority of the pixels were in the black background, which comprises low gray levels, while the brighter NMJ pixels were at a higher gray level. The software calculated the point along the x-axis at which the histogram could be split into two groups so that the summed variance of the distributions on each side of the split was minimized. After the software set the threshold levels, the pixels above those levels were categorized as positive and counted. The software used this method to categorize and count the pixels as being stain 1, stain 2, or both for each pixel in the NMJ.

**Figure 1:**
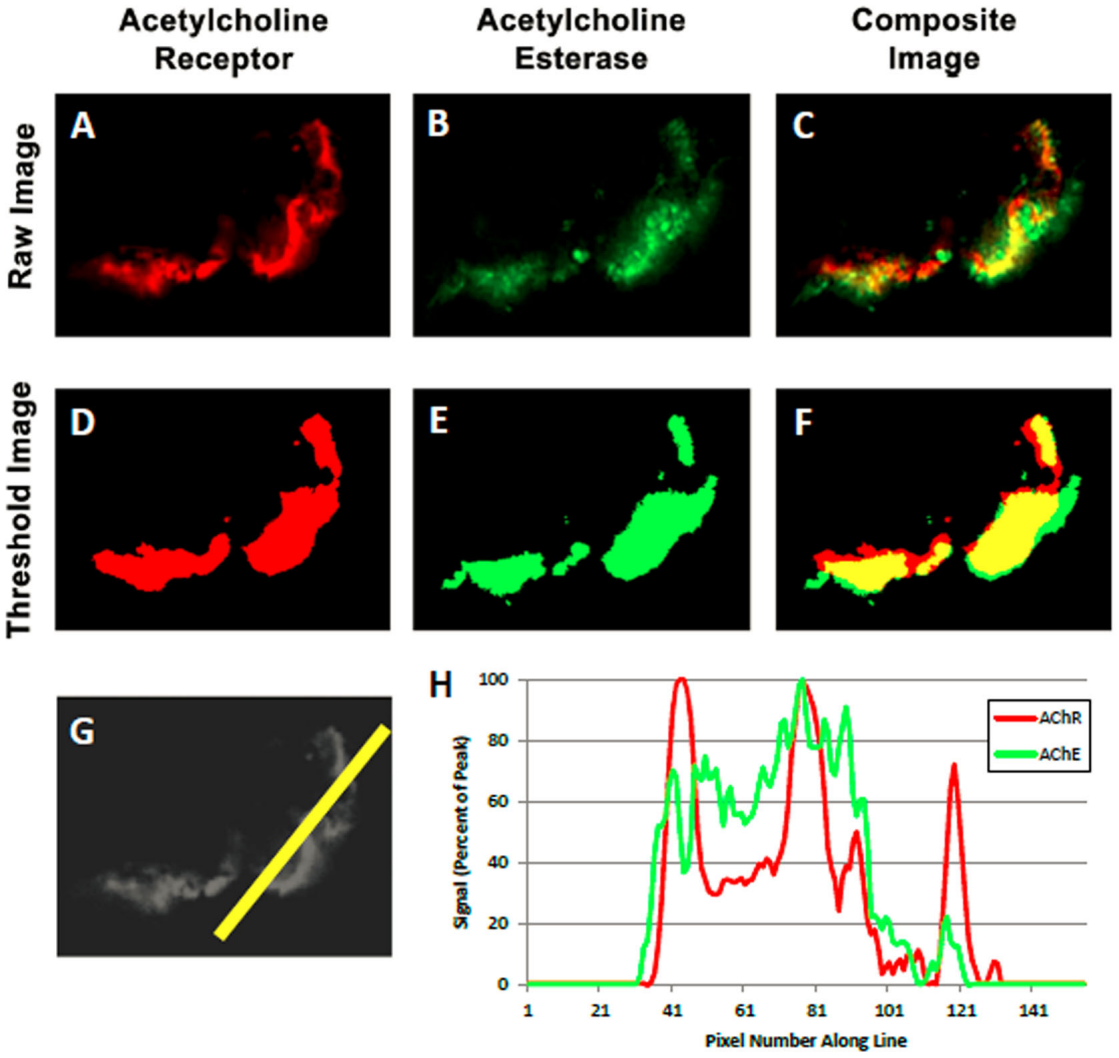
Determination of appositional score. Sample images are shown for a single NMJ from a *spinalis* biopsy of a child with CP. The distribution of AChR (Panel A), AChE (B), and the composite image of both AChR and AChE (C) are shown. Each image was thresholded using the standard algorithm provided in the Image Pro Software, which objectively identifies foreground versus background pixels based on the distribution of intensities in the image, resulting in images D, E, and F. Distinct boundaries were reproducibly identifiable relative to background using this approach. A line drawn through the NMJ (G) provided the corresponding histogram (H) which displayed the intensities of AChR and AChE staining across the NMJ. The pixels above the preset threshold levels were categorized and counted to determine appositional scores.

The relative distributions of AChR, AChE, and either SV2 or β-laminin were compared pair-wise in a two-step process that used (i) the Image Pro Plus thresholding algorithm to provide an objective assessment of positive staining based on an iterative analysis of an image's histogram to find the best split between foreground and background signal, followed by (ii) a customized software macro to calculate an appositional score indicating the degree of non-colocalization (see Text S1 and **Figure 1**). Once the analyses were complete, the blind was broken. Data calculated for NMJs from IS patients were used to establish control values and ranges and compared to those from CP patients to assess statistical differences in relative NMJ component distribution.

### Histological Assessment of Muscle Fibers

A subset of samples (n = 10 patients with CP; 10 control patients with IS) were stained using a clinical-diagnostic method to detect myosin ATPase [21]. Briefly, 8 µm thick sections were stained at high (10.2) or low (4.65) pH to distinguish type I and type II fibers, which were counted manually in sequential fields under an Olympus BX-60 microscope using a 10× objective. Data were acquired using Image Pro Plus software. A minimum of 100 fibers were counted for each patient sample.

### Western Blot Analysis

Myosin heavy chain (MYH) isoforms, of which there are at least 10 Class II sarcomeric types with three that predominate in mature skeletal muscle [22], were analyzed by Western Blotting (n = 3 patients with CP; 4 control patients with IS) using monoclonal antibodies directed against subsets of the Class II MYHs. Antibodies utilized included the monoclonal antibodies in table 1 (all from the Developmental Studies Hybridoma Bank, Ames, IA).

**Table 1:** Monoclonal Antibodies to Myosin Heavy Chains.

Monoclonal Designation	Myosin Type Recognized	Gene Symbol
A4.74	Fast Types IIx, IIa, embryonic, and IIb	MYH1, MYH2, MYH3, & MYH4
A4.951	Slow Type I	MYH7
F1.652	Embryonic	MYH3

Antibodies were purified from hybridoma media using Protein G agarose columns (Invitrogen, Carlsbad, CA) following the manufacturer's specifications and validated on control blots prior to use. For blotting, protein samples were separated using standard polyacrylamide gel electrophoresis protocols with pre-cast Novex gels and pre-made buffers (all from Life Technologies/Invitrogen/Novex). Bands were transferred to PVDF membranes using a Novex transfer apparatus and buffers and detected using Enhanced Chemi-Luminescence detection kits with HRP-conjugated anti-mouse secondary anitbodies (Life Technologies/Invitrogen/Novex).

### Real-time RT-PCR

Total RNA was extracted from frozen tissue sections (n = 12 patients with CP; 12 control patients with IS) using Trizol (Invitrogen). Complementary DNA synthesis was carried out using iScript cDNA synthesis kits (BioRad), and real-time PCR was performed with iQ SYBR Green Supermix (Biorad) on a MyIQ thermocycler (BioRad) to probe gene expression patterns. Primers for acetylcholine receptor (AChR) subunits α1, α7, δ, ε, and γ as well as β1 and β2 syntrophin, myogenin, and AChE were used; β–glucuronidase (GUSB) was selected as a normalization control based on its consistent expression across samples (**Table S2**). PCR conditions were defined as follows: 5 min at 94°C followed by 30 cycles of 30 s at 94°C, 75 s at 55°C, 45 s at 72°C, and one cycle of 72°C for 7 min.

#### Telomere Length Analysis

Telomere lengths were estimated using a quantitative PCR method [23] with slight modifications (Text S1) and telomere to single copy gene (T/S) ratios were calculated (n = 10 patients with CP; 10 control patients with IS) relative to a normal leg muscle sample using the Pfaffl method [24].

### Analysis of Transmission Electron Microscopy (TEM) Images

Samples were prepared for TEM analysis based on previously described procedures elaborated in Text S1 [25]. Five distinct contact regions were analyzed for each NMJ; contact regions were defined based on the presence of a distinct synaptic gutter. Morphometric differences were determined for three measures: fold length, distance between folds, and cross sectional area of mitochondria. The portion of the nerve terminal within the synaptic gutter was specifically evaluated for each contact region using ImageJ software [26]. The distance between folds was measured as the distance along the synaptic cleft between the neighboring edges of two successive folds. Fold length was measured as the distance from the synaptic cleft to the furthest region each contiguous fold extended into the muscle following a previously established method [27]. Mitochondria at the nerve terminal was estimated as the cross-sectional area of mitochondria per square micron area of the nerve terminal present. Each measurement approach was validated using inter-rater reliability scores determined by comparing assessments from two independent, blinded investigators (see **Table S3**).

### Immunofluorescence Analysis of Mitochondrial Content

Sections were brought to room temperature and held in 0.1 M PB. Slides were blocked with 3% BSA (Sigma) for 1 hr and incubated overnight with mouse monoclonal antibody diluted 1∶200, for complex VI subunit I (Mitosciences, Oregon). Slides were rinsed 3× with PB and then incubated with anti-mouse Alexa Fluor-488 (Invitrogen) diluted 1∶400, in a solution containing 0.16 µg/mL α-bungarotoxin-AlexaFlour™ 555 for 1 h. The sections were rinsed 3×, 10 min each with PB and mounted in PB on slides with a 22×40 mm coverslip for visualization. Confocal Z-stacks were acquired with a 40× C-Apochromat water immersion lens (Numerical Aperture 1.2) on a Zeiss LSM510 microscope using identical excitation, emission, and acquisition settings for all samples.

## Results

### Screening of NMJ Probes

To identify suitable antibody probes for phase one of the study, antibodies to critical NMJ components were assessed for their detectability by indirect immunofluorescence in control human skeletal muscle tissue. Sections were double labeled with α-bungarotoxin and the indicated probe. As summarized in **Table S4**, probes were rated as easily detectable over background, faint, very faint, undetectable, or non-specific relative to α-bungarotoxin. Sixteen antibodies were screened for 15 different NMJ markers; only three were judged to be both readily detectable over background based on a standard thresholding algorithm (built into Image Pro Plus) and localized to NMJs as defined by α-bungarotoxin. These three were the only antibodies used in the subsequent study.

### Disruption of NMJ Components in Children with Spastic CP

To evaluate potential NMJ disruptions, the relative localization of critical NMJ proteins was evaluated by blinded investigators using wide-field fluorescence microscopy. Since AChR and AChE are important and well-known participants in the initiation (AChR) and termination (AChE) of nerve-induced muscle contraction [11], [28], these proteins were included in all comparisons. AChR localizes to the postsynaptic muscle membrane; whereas, AChE localizes to the synaptic basal lamina. In addition, SV2 and laminin β2 distributions were analyzed based on the antibody screening. SV2 is a transmembrane keratan sulfate proteoglycan expressed at nerve endings and routinely used as a presynaptic marker for NMJs. Laminins are trimeric proteins containing α, β, and γ chains and providing both structural support and signaling within basal lamina [29], [30], [31].

**Figure 2:**
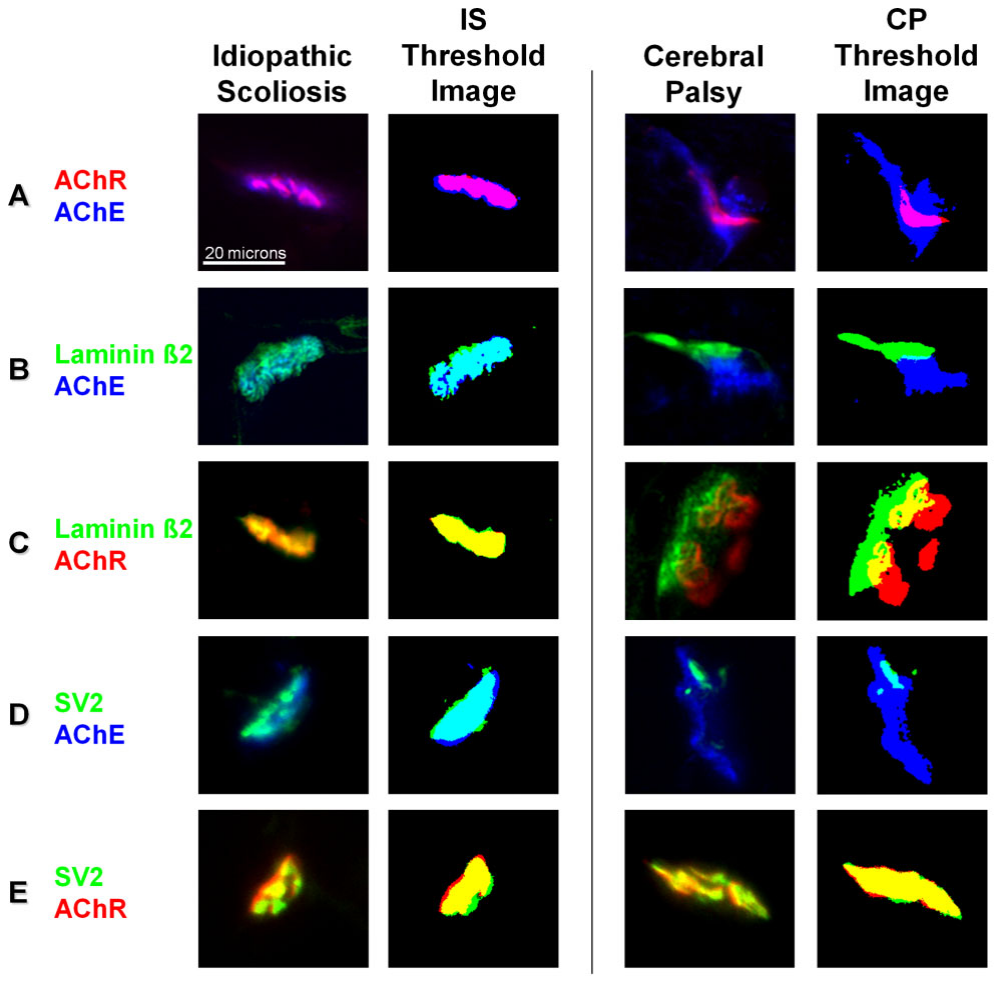
Typical staining patterns for idiopathic scoliosis (IS) and CP patients with the corresponding threshold images. *Spinalis* samples were triple stained for AChR (red), AChE (blue), and either laminin β2 or SV2 (green). Representative staining patterns with two of the three stains are shown. Compared to idiopathic scoliosis patients, CP patients generally had more AChE present outside AChR (A), more AChE present outside laminin β2 (B), less laminin β2 present outside AChE (B), more AChR present outside laminin β2 (C), and more AChE present outside SV2 (D). Both CP and IS samples had a similar distribution of SV2 relative to AChR (E).

Data from immunofluorescence assays were included in statistical comparisons when a minimum of 15 distinct NMJs per patient were available for each staining combination. A total of 1899 NMJs were examined from IS patients and 1589 from CP patients. Representative staining for NMJs from IS and CP patients showing the differences in relative distribution for each pair of proteins is shown in **Figure 2**. The approach to quantifying co-localization between probes is illustrated in **Figure 1**, which shows how the thresholding algorithm was used to define the limits of staining at NMJs and used to calculate overlap in staining patterns.

The limits of each staining pattern were determined using an objective thresholding algorithm, and the degree of overlap between two staining patterns was calculated using a custom software macro that compared thresholded images on a pixel by pixel basis. Data were calculated as the proportion of one thresholded staining pattern that was present outside the other and are reported as colocalization scores such that a score of zero indicates complete overlap and a score of 1 indicates no overlap.

Due to the hierarchical nature of the data acquired (*i.e*., there were two patient groups with multiple subjects in each group with each subject having multiple NMJs analyzed), nested statistical analyses were carried out. First, all NMJs from all children with a particular diagnosis were pooled into two groups (1589 CP and 1899 IS NMJs) and compared. Second, a single co-localization score was calculated for each patient and these scores were compared between the two groups (25 CP and 28 IS patients). Finally, data from the entire pool of control/IS patients were pooled and values from each CP patient were compared to this group one at a time. In this way, we were able to evaluate differences in the appearance of NMJs within CP and IS patients at multiple different levels.

Comparison 1. All NMJs from each group were pooled and compared directly; this approach disregards patient to patient variability. Both groups had very well matched SV2 versus AChR; however, CP patients had significantly more AChE present outside AChR, AChE present outside laminin β2, AChR present outside laminin β2, and AChE present outside SV2. In addition, there was statistically less laminin β2 present outside AChE in the NMJs from CP patients. (see **Table 2**).
Comparison 2. A median co-localization score was calculated for the set of values determined for each child; with this approach, variability from within each patient was not included since data from each patient were represented by a single value. Comparison of the median scores between the two patient groups (see **Table 3**) indicated that only AChE outside of laminin β2 was significantly higher in the CP patients (p = 0.017 by Mann-Whitney U test).
Comparison 3. Each CP patient was compared to the pooled data from all control/IS patients in order to determine how the set of NMJs from each CP patient may have differed from the entire control population of IS patients. Pairwise Mann-Whitney tests were applied with Bonferroni corrections employed to account for multiple comparisons. The results are shown in **Table 4**. Twenty-one of the 25 CP patients (84%) had at least one comparison that was significantly different from the pooled IS group. The difference that arose most frequently was AChE outside of laminin β2. Overall, however, it was difficult to discern a pattern of disruption across patients. Accordingly, a logistic regression analysis was employed to determine which, if any, measures were actually predictive of a CP diagnosis. The one measure that was found to be significantly predictive of a diagnosis of CP was AChE outside of laminin β2 (p = 0.021).

**Table 2:** Median values and ranges for NMJ component comparisons; patients pooled by diagnosis.

Comparison	Idiopathic scoliosis		Cerebral palsy		Mann-
	(n = 1899 NMJs)		(n = 1589 NMJs)		Whitney
	Median	Range	Median	Range	p value
ROE	0.04	0.00–0.80	0.04	0.00–1.00	0.154
EOR	0.30	0.01–1.00	0.32	0.00–1.00	0.002*
LOE	0.39	0.05–0.92	0.36	0.00–1.00	0.002*
EOL	0.07	0.00–0.84	0.10	0.00–1.00	0.000*
LOR	0.51	0.08–0.90	0.50	0.12–0.90	0.141
ROL	0.04	0.00–0.79	0.05	0.00–1.00	0.000*
VOE	0.07	0.00–0.64	0.07	0.00–1.00	0.544
EOV	0.55	0.15–1.00	0.57	0.06–1.00	0.022*
VOR	0.20	0.00–0.67	0.20	0.00–0.80	0.216
ROV	0.45	0.00–1.00	0.46	0.00–1.00	0.955

R = AChR; O = outside of; E = AChE; L = laminin β2; V = SV2.

* represents p values<0.05 by Mann-Whitney.

**Table 3:** Median values and ranges for NMJ non-colocalization scores; each patients received a score equivalent to the median values within their NMJs.

	Idiopathic scoliosis		Cerebral palsy		Mann-
Comparison	(n = 28 patients)		(n = 25 patients)		Whitney
	Median	Range	Median	Range	p value
ROE	0.04	0.02–0.07	0.04	0.01–0.08	0.487
EOR	0.30	0.24–0.44	0.31	0.20–0.43	0.539
LOE	0.41	0.20–0.56	0.38	0.19–0.49	0.170
EOL	0.06	0.02–0.19	0.10	0.01–0.23	0.017*
LOR	0.53	0.35–0.69	0.51	0.34–0.64	0.226
ROL	0.04	0–0.16	0.05	0.01–0.16	0.349
VOE	0.07	0.03–0.13	0.06	0.01–0.22	0.964
EOV	0.54	0.33–0.69	0.58	0.39–0.76	0.121
VOR	0.19	0.11–0.43	0.21	0.10–0.40	0.838
ROV	0.44	0.31–0.62	0.45	0.26–0.69	0.972

R = AChR; O = outside of; E = AChE; L = laminin β2; V = SV2.

* represents p values<0.05 by Mann-Whitney.

**Table 4:** Comparison of individual CP patients with the idiopathic scoliosis group.

CP Sample	ROE	EOR	LOE	EOL	LOR	ROL	VOE	EOV	VOR	ROV
RA003			−−−							
RA006	+++	−−−								
RA010		+++		−−−						
RA013	+++	−−−				+++		+++		+++
RA015								−−−		−−−
RA016										
RA019			−−−		−−−					
RA026	+++						+++			
RA028	−−−							−−−		−−−
RA030	−−−	+++		+++	+++		−−−			
RA031			−−−							
RA032	−−−		−−−		−−−					
RA035	−−−			+++						
RA040		+++		+++				+++		
RA041		+++	+++	+++	+++	+++			+++	
RA042		+++		+++			+++			
RA046				+++						
RA052										
RA053		−−−	−−−		−−−			+++		+++
RA059				+++		+++				
RA062										
RA069					−−−					
RA100	+++									
RA104										
RA111				+++						

Pair-wise Mann-Whitney tests were run to determine how individual CP patients compared to the idiopathic scoliosis groups. R = AChR; O = outside of; E = AChE; L = laminin β2; V = SV2. +++ indicates that the child had significantly higher values than the control group, while −−− indicates that the child had significantly lower values than the control group (p≤0.002; note that this level of significance was selected because there were 25 CP children compared to the control/IS group).

The results of these comparisons indicate that the NMJs in the CP patient group were disrupted with the disruption that was the most highly affected, that appeared in the greatest proportion of patients, and that was most predictive of a diagnosis of CP being AChE appearing outside the limits of laminin β2 staining.

### Neuromotor Maturational Status of Children with Spastic CP and Idiopathic Scoliosis

To assess the maturational status of muscle from the CP and IS groups, a series of indicative assays was carried out including histomorphometry of type I and type II muscle fiber-types, Western blotting for embryonic myosin heavy chain, quantitative real time PCR of developmentally-regulated genes, and telomere length analysis.

**Figure 3:**
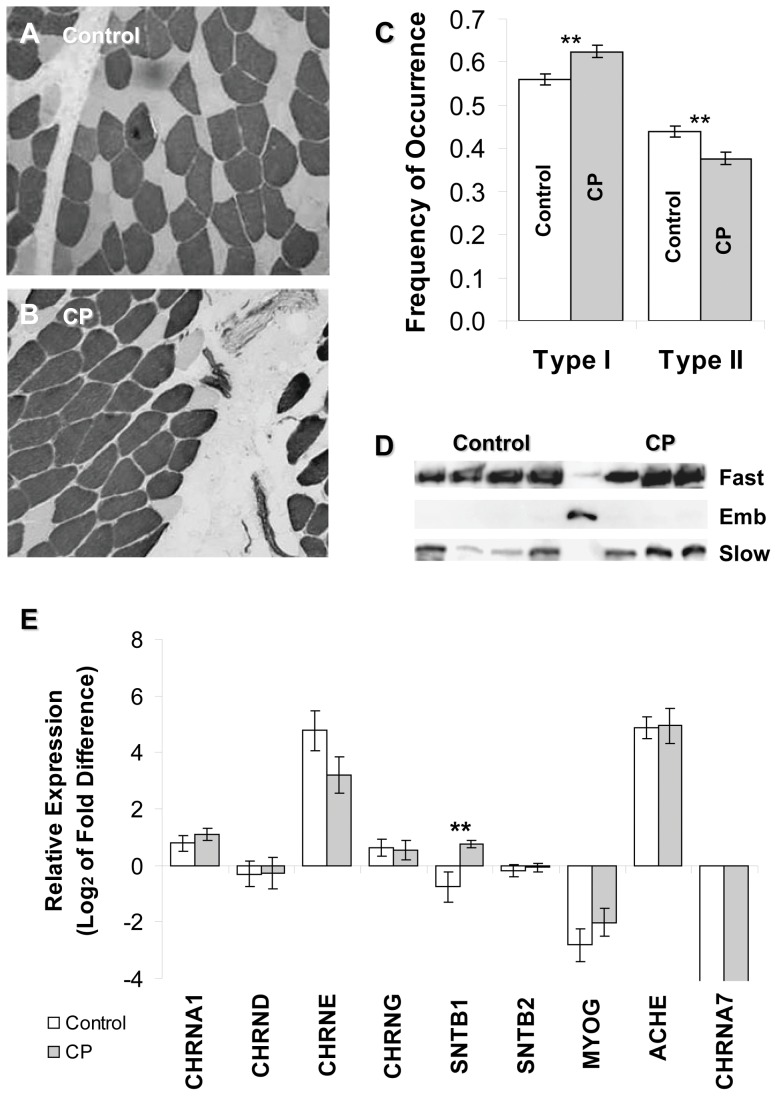
Comparison of neuromotor maturation in idiopathic scoliosis (IS) and CP patients. A) *Spinalis* sample from a control patient showing distinctly stained type I (dark) and type II (light) fibers using an ATPase assay at low pH (4.65). B) *Spinalis* sample from a patient with CP showing type I and type II fibers with an overall mature appearance similar to that seen in panel A, except for an increased predominance of type I fibers. C) Analysis of fiber typing: at least 100 fibers were enumerated as either type I or type II fibers for each patient sample (n = 10 per group) and compared for fiber type predominance. The average frequency of each type is shown. Patients with CP had a significantly higher frequency of type I fibers than patients with IS (p = 0.013 by Mann-Whitney) and a significantly lower frequency of type II fibers (p = 0.044 by Mann-Whitney). D) CP (lanes 6–8) and IS (lanes 1–4) muscles were analyzed for the presence of fast, slow, and embryonic MYHs by Western blot. Fetal muscle (lane 5) was used as a positive control for immaturity and the presence of embryonic MYH. Antibodies used are as follows: A4.74 (fast) for MYH1, MYH2, and MYH4; F1.652 (embryonic) for MYH3; A4.951 (slow type I) for MYH7. Neither CP nor control muscle contained the embryonic MYH isoform. E) RNA was extracted from *spinalis* muscle and subjected to real time RT-PCR. Data are presented as mean (± SD) fold differences compared to fetal tissue (n = 12 CP, 12 IS). AChRα1 (CHRNA1), AChRδ (CHRND), AChRε (CHRNE), AChRγ (CHRNG), AChRα7 (CHRNA7), myogenin (MYOG), AChE (ACHE), and β2 syntrophin (SNTB2), did not show significant differences between the CP and control groups. β1 syntrophin (SNTB1) expression was significantly higher in CP samples than control samples (p<0.001 by Mann-Whitney).

Comparison 1. The distribution and conformation of muscle fiber types were evaluated using a clinical-diagnostic ATPase histochemistry approach, which is related to neuromotor development and innervation [32]. Staining patterns were generated by ATPase reactions carried out at high and low pH to reveal fast and slow fibers, respectively, in the context of tissue organization. The overall appearance of muscles from patients with IS (**Figure 3A**) and CP (**Figure 3B**) were generally similar and both differed substantially from immature muscle fibers, which do not evince clear fiber type patterning. Although similar in shape and size across samples, the CP samples tended to have thicker endomysium (**Figure 3B**) and higher levels of fatty deposition (not shown). These observations are consistent with earlier reports that muscle from children with CP is significantly stiffer than muscle from typically developing children in association with endomysial thickening and an increase in ECM content [33] and that children with CP have a greater adipose tissue infiltration of skeletal muscle than typically developing children [34].
  Although the muscle fibers from CP and IS patients were similar, there was a slight predominance of type I over type II fibers in CP patients (**Figure 3C**), which is consistent with previous reports [33], [35], [36]. A predominance of type I fibers has been associated with myopathic diseases and dystrophies and may be related to alterations in the use patterns for the specific muscle [37], [38]. Differences in the appearance of fast type IIx, IIa or mixed type fibers were not evaluated in the present study; however, we found no significant difference in NMJ disruption scores associated with slow (i.e., MAb A4.951 positive) versus all fast (i.e., MAb A4.951 negative) muscle fiber types by Mann-Whitney U test (p>>0.05; n = 80 fast fiber NMJs and 104 slow fiber NMJs); thus, the occurrence of disrupted NMJs appeared unrelated to fast versus slow muscle fiber type. Additionally, there was no significant correlation between the degree of NMJ disruption assessed by median AChE outside of laminin β2 and the ratio of type I to type II fibers present in each child's biopsy material (Spearman's rho = −0.071).
  Overall, the results of Comparison 1 indicate that the maturational status of the muscle in the two patient groups was similar although there were notable differences in the appearance of the muscle fibers that may warrant further investigation.
Comparison 2. The accumulation of the MYH gene products in muscle changes during development and neuromotor maturation [22]. Accordingly, we evaluated the MYH protein content of a small number of samples (owing to the large amount of tissue needed to carry out the analysis, we were not able to perform these assays on all samples). In particular, immature muscle expresses embryonic myosin (MYH3) [39], and we used antibodies that recognize embryonic, adult slow (MYH7) and a combination of fast MYH isoforms (MYH1, MYH2, MYH3, and MYH4) to evaluate the presence of developmental MYH3 in CP and IS samples. Gestational tissue was used as control for MYH3. As seen in the Western blots shown in **Figure 3D**, CP and control samples had similar expression patterns such that fast and slow isoforms were expressed, albeit at apparently different ratios, but that the embryonic MYH3 isoform was not found regardless of diagnosis. These data suggest that *spinalis* from the CP group was not substantially delayed in neuromotor maturation relative to controls and that the tissues were not regenerating at the time of surgery.
Comparison 3. The gene expression profiles of maturing muscle have been well studied, and several genes exhibit differential expression during neuromotor development including the γ- and ε-subunits of the AChR, myogenin, and β1 syntrophin [11], [13], [15]. Accordingly, real-time quantitative PCR was employed to estimate relative mRNA levels of molecular markers for neuromotor maturation in CP and control/IS muscle. Transcript levels for the AChR subunits α1, α7, δ, ε, and γ were determined (**Figure 3E**). AChRγ is expressed from the CHRNG gene in immature and in denervated muscle; it is replaced by AChRε (CHRNE) in mature/innervated muscle [11]. The levels of AChRγ mRNA were similar in the CP and IS groups. AChRα7 (CHRNA7), which can bind α-Btx like AChRα1 (CHRNA1) [40], is expressed developmentally and in chronically denervated [41] or pathologic muscle [42], was found in fetal tissue but was undetectable in any patient sample. Similarly, myogenin was not significantly different between the CP and IS groups but was significantly elevated in the fetal control. These results suggest that both groups attained a similar level of maturation.
  Interestingly, β1 syntrophin (SNTB1), which is normally down-regulated in mature muscle fibers, was significantly elevated in CP samples. SNTB1 transcripts can be found in blood vessels, nerves, and fibrous tissue [15], and given the lack of other molecular markers for immaturity, the elevation of SNTB1 in CP samples may actually reflect the increased fibrosis and fatty-content of CP muscle samples noted in Comparison 1.
  Overall, the gene expression patterns evaluated were consistent with the CP and IS populations having attained a similar level of muscle maturation. In addition, there were no significant correlations found between median AChE outside of laminin β2 and the relative expression level of any gene examined (Spearman's rho <0.6 for each gene evaluated).
Comparison 4. Telomere length indicates the proliferative capacity and regenerative history of myoblasts with older or multiply-regenerated muscle fibers having shorter telomeres [43], [44]. To determine whether muscle from CP and control patients had undergone the same degree of development and regeneration, a quantitative PCR method [23] was used to assess telomere length (**Table 5**). No significant differences were seen in the ratio of telomere to single copy gene (T/S ratio) between patients with IS and CP. In addition, no correlation was found between median AChE outside of laminin β2 and the relative telomere length in the CP population (Spearman's rho = 0.224). Interestingly, both patient groups had T/S ratios between the normative adult muscle control and three DMD patients, which were analyzed as positive controls for reduced telomere length. The DMD patients had lower T/S ratios than both the IS and CP patients, but both groups had T/S ratios lower than expected relative to adult controls. These results suggest that scoliosis, but not CP, may be associated with increased regenerative cycling in *spinalis* muscle and support the notion that CP and IS patients had attained a similar level of neuromotor maturation.

**Table 5:** Telomere lengths expressed as T/S ratio relative to normal leg muscle in patients with CP, idiopathic scoliosis, or DMD.

Subject	Diagnosis	T/S Ratio Relative to Normal	Median EOL	Subject	Diagnosis	T/S Ratio Relative to Normal	Median EOL	Subject	Diagnosis	T/S Ratio Relative to Normal
RA014	IS	0.95	0.023	RA003	CP	0.74	0.069	DMD1	DMD	0.37
RA017	IS	1.07	0.027	RA016	CP	0.98	0.054	DMD2	DMD	0.61
RA021	IS	0.94	0.161	RA019	CP	0.75	0.081	DMD3	DMD	0.78
RA022	IS	0.55	0.047	RA026	CP	0.89	0.065			
RA024	IS	1.24	0.084	RA030	CP	1.00	0.161			
RA027	IS	0.94	0.094	RA041	CP	0.91	0.226			
RA103	IS	0.51	0.044	RA042	CP	0.83	0.234			
RA105	IS	1.01	0.076	RA100	CP	0.70	0.081			
RA113	IS	0.79	0.070	RA104	CP	0.67	0.054			
RA114	IS	0.81	0.067	RA111	CP	0.57	0.144			
**IS Mean**		**0.88**		**CP Mean**		**0.80**		**DMD Mean**		**0.59**

T/S Ratio Relative to Normal.

The results of these four sets of assessments are all consistent with neuromotor maturation having progressed to a similar extent in the CP patient and control/IS groups. To further verify this result, we performed an additional phase of studies utilizing transmission electron microscopy.

### Transmission Electron Microscopy (TEM) of NMJs

**Figure 4:**
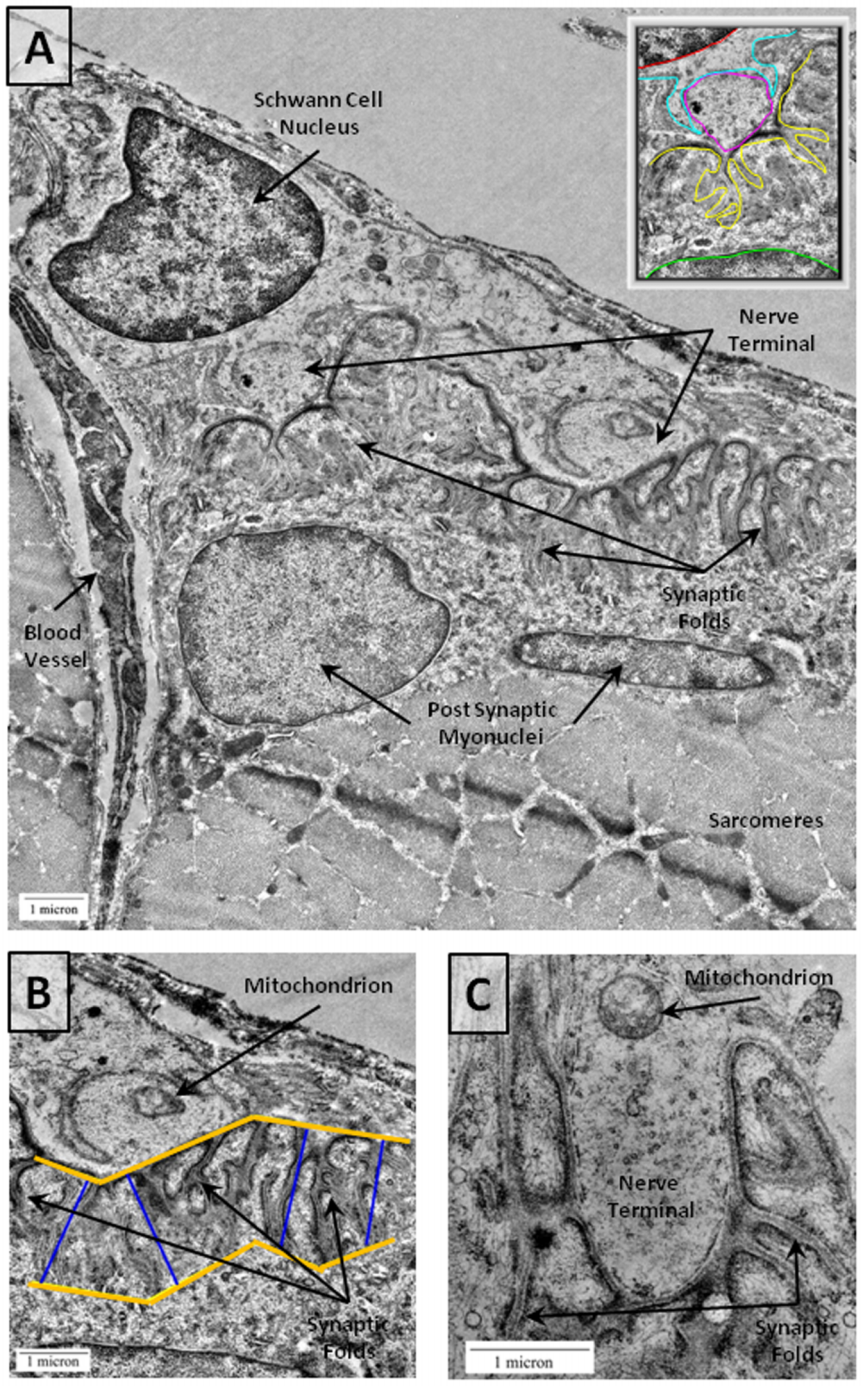
NMJs from CP Patients by Electron Microscopy. A) Top panel shows an electron micrograph of a CP NMJ. A zoomed out image is shown in the upper right corner with a red line marking a Schwann cell nucleus, cyan marking the Schwann cell membrane, magenta marking the nerve terminal, yellow marking the postsynaptic folds, and green marking the postsynaptic nucleus. Scale bars = 1 µm. B) A zoomed image of primary folds in the CP NMJ is also shown. The blue lines indicate the general depth of postsynaptic fold penetrance into the muscle. For data analysis, each fold was measured separately using ImageJ software to draw trace lines for individual folds following the methods outlined by Banks et al. [27]. C) A CP NMJ with a single mitochondrion in the nerve terminal (arrow). The total area occupied by mitochondria was calculated with ImageJ software by tracing each mitochondrion segment and measuring the summed mitochondrial area relative to the total area of the terminal.

**Figure 5:**
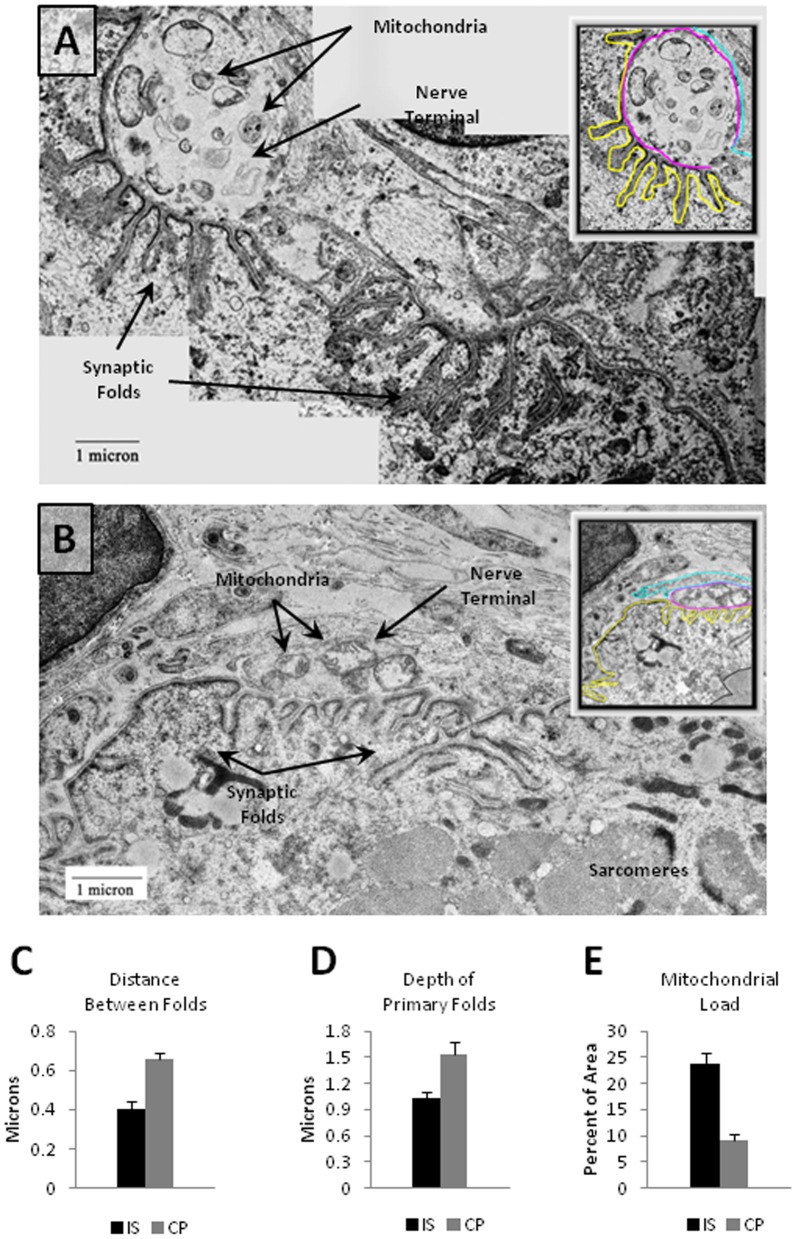
CP NMJs have a greater distance between postsynaptic folds, longer postsynaptic folds, and reduced mitochondrial load. A,B) Two examples of control NMJs with zoomed out images in the corners. The cyan lines label Schwann cell membrane, magenta lines label the nerve terminals, and yellow lines label the postsynaptic membranes. Note the presence of mitochondria in the nerve terminals. C) Graph depicting the average distance between folds in CP (0.655 µm±0.0368 SEM, n = 25 NMJs) as compared to controls (0.392 µm±0.0299 SEM, n = 25 NMJs). There was a statistically significant (p<0.0006) increase in the distance between primary folds in CP NMJs as compared to control NMJs. D) Graph indicating the mean length of primary folds in CP and control sample in microns. Mean primary fold length in CP (1.536 µm±0.128 SEM, n = 25 NMJs) was significantly (p = 0.0062) higher in CP as compared to controls (1.012 µm±0.0636 SEM, n = 25 NMJs). E) Graph depicting a significant (p = 0.0001) reduction in mitochondrial area per square micron of the nerve terminal is evident in CP (7.8%+1.8 SEM, n = 25 NMJs) as compared to control samples (23.8%±1.4 SEM, n = 25 NMJs). Error bars in all the graphs are +/−SEM.

Ultra-structural analysis using TEM was undertaken to examine the degree of structural disruption in NMJs of both CP and IS patients. Samples from both groups appeared generally similar with closely apposed nerve and muscle and no evidence of polyneuronal innervation at the EM or light microscopy level. NMJs from CP patients had pronounced postsynaptic folds and postsynaptic myonuclei consistent with mature NMJs (**Figure 4**). Interestingly, both the level of presynaptic mitochondrial content and the extent of postsynaptic folding appeared to differ in the two groups with CP patients exhibiting what appeared to be deeper postsynaptic folds and fewer presynaptic mitochondria (**Figure 5** **and** **6**). To evaluate these differences, a series of measurements to characterize synaptic folding and the distribution of mitochondria were performed. Measures were validated (see **Table S3**) by comparing results from two independent raters; the measures were found to be highly reliable.

**Figure 6:**
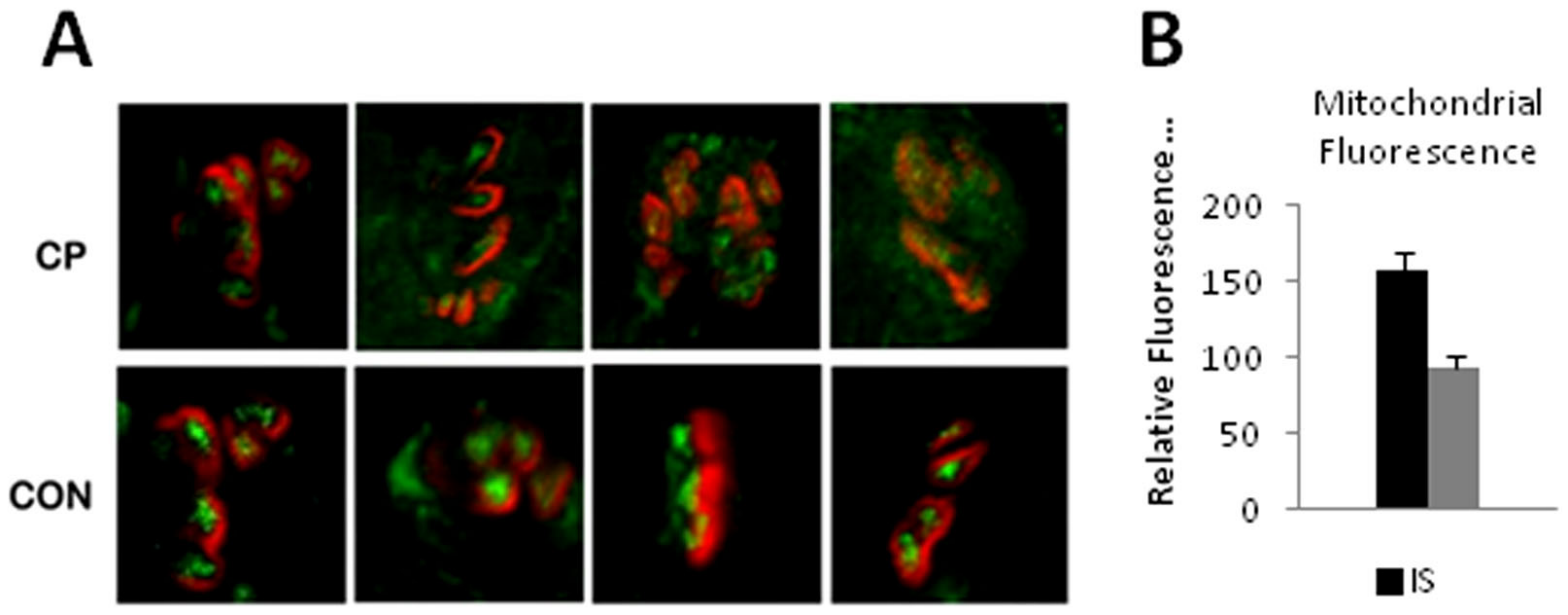
CP NMJs have a reduced mitochondrial load. A) Confocal images showing double staining for acetylcholine receptor by α-bungarotoxin (red) and mitochondria (green) using a mouse monoclonal antibody for complex VI subunit I of cytochrome c oxidase (Mitosciences, Oregon). Images represent the mean intensities of fluorescence signal across 10 z-stacked images by confocal microscopy. CP NMJs show a decreased intensity for synaptic mitochondria. B) Average mitochondrial fluorescence intensities of NMJs of CP (93.25±8.017 SEM, n = 25 NMJs) and control (156.70±12.035 SEM, n = 25 NMJs) samples. Bars represent the mean ± SEM of immunofluorescence intensity in arbitrary fluorescence units based on the signal from the mitochondrial stain at the NMJs of CP and control samples.

Comparison 1. The number and length of postsynaptic junctional folds were found to differ between the two groups. There was a statistically significant increase in the length of primary postsynaptic folds between CP NMJs (**Figure 4A, 4B**) and IS NMJs (**Figure 5A and 5B**). There was a significantly greater distance between synaptic folds in CP as compared to control samples (p = 0.0006; **Figure 5C**). In control muscle samples, the postsynaptic membrane showed infoldings every 0.392±0.029 µm, versus CP showing folds every 0.656±0.0368 µm. Folds in CP NMJs, were also approximately 1.5 times deeper than folds in the IS/control samples (**Figure 5D**).
Comparison 2. The distribution of mitochondria within the synaptic gutter of nerve terminals differed in the two groups. Mitochondria are important for normal functioning of the NMJ and cluster at nerve terminals [45], [46] presumably to provide the high level of ATP needed to support axonal transport, synaptic vesicle formation, purinergic signaling, and ion pump activity [47]. We observed an apparent reduction in the number of mitochondrial regions (**Figures 4C** **,** **5A, and 5B**), and the proportional cross-sectional area of mitochondria in the nerve terminal within the synaptic gutter was significantly lower (p = 0.0001; **Figure 5E**). To further assess the mitochondrial composition of NMJs, additional samples were double stained for AChR using fluorescent bungarotoxin and for mitochondria using a mouse monoclonal antibody to complex VI subunit I (Mitosciences, Oregon). Specimens were imaged under identical conditions to obtain confocal Z stacks (**Figure 6A**). The fluorescence emission through the nerve terminal region of the z stacks was calculated using Image J software and found to be significantly lower in CP samples (93.25±8.02, n = 25 NMJs) as compared to controls (156.68±6.02 SD, n = 25 NMJs) (**Figure 6B**).

These two sets of comparisons indicate that NMJs from the two patient groups were similar with a generally mature appearance on ultra-structural examination. Differences in the distribution of presynaptic mitochondria and the conformation of post synaptic folds suggest dysmorphism in the CP group at the level of postsynaptic folding and presynaptic organelle distribution.

## Discussion

### Microanatomic Disruption of NMJs

Our results indicate that neuromotor synapses from erector spinae muscle of patients with CP may be structurally dysmorphic and provides details about deformations present in a population of children with spastic CP. In particular, the localization of both AChE and laminin β2, which are critical components of the synaptic basal lamina, appear disrupted in CP on light microscopic examination. Transmission electron microscopy indicates that differences in the conformation of postsynaptic folds and the reduction of mitochondria in presynaptic nerve endings are also associated with spastic CP. These microanatomic findings extend previous reports suggesting NMJ disruption [6], [7], [9], [10]. In addition, our data suggest that muscle samples from CP and control patients with IS had attained similar levels of maturation and retained similar levels of regenerative capacity (i.e. telomere lengths). Thus, NMJ disruption in CP appears unrelated to neuromotor maturational status.

The presence of disrupted motor synapses in patients participating in this study is surprising as NMJs from mature muscle normally have very well defined and organized pre-, post-, and synaptic components [11]. Our data suggest that intrinsic abnormalities involving the synaptic basal lamina may be prevalent in CP muscle with the abnormality that was most common, most significant, and most likely to be associated with a diagnosis of CP being the localization of AChE outside laminin β2. Intriguingly, despite these differences in the NMJs from CP and control groups, some structural aspects of NMJs were nearly identical in the two groups. For example, the presence of SV2 outside of AChR and AChR outside of SV2 were not significantly different between the patient groups (p = 0.216 and 0.995, respectively), indicating that the pre- (SV2) and post-junctional (AChR) components may be well aligned in CP patients. Dysmorphism in CP, therefore, lies within the synaptic cleft represented by the junctional components AChE and laminin β2. Interestingly, our results also suggest that either laminin β2, which mediates pre- and postsynaptic organization [29], functions properly to establish CP NMJs or that another molecule compensates for laminin β2 dysregulation.

The localization and functional involvement of AChE and laminin β2 in NMJs are critical to NMJ structure and efficient neurotransmission. Although specific data on human NMJs are limited, in the model systems that have been studied, AChE is expressed by junctional myonuclei in response to nerve activity and myofiber depolarization with the resulting AChE clustered at the NMJ as a result of neural agrin signaling [48]. The asymmetric form of AChE that predominates at NMJs comprises three tetramers of catalytic subunits linked to a collagen-Q tail [5], [28]; the ColQ C-terminus binds muscle-specific tyrosine kinase (MuSK) in the postsynaptic membrane [49] while the tail portion of the ColQ binds perlecan, which accumulates in the synaptic basal lamina and is itself associated with α-dystroglycan in the muscle membrane [28]. In short, the regulation of AChE expression, protein clustering mechanisms active at NMJs, and linkages between AChE and other NMJ components drive its normal localization to the basal lamina. Laminin β2, on the other hand, is a subunit of the heterotrimeric laminin proteins that are critical in the formation and function of basal lamina [30]. Laminin β2, which is enriched in the basal lamina of human [50] and rodent [29] NMJs, is produced by the muscle, and plays an autocrine role in promoting proper NMJ maturation [29]. In rodents, laminin β2 has been suggested to stop outgrowth of neurites from motor neurons [51], to bind voltage-gated Ca^2+^ channels and induce presynaptic differentiation of motor nerve terminals and organization of active zones *in vitro*, [29], [52], [53], and to prevent Schwann cell processes from entering the synaptic cleft [54]. Interestingly, laminin β2 mRNA levels are elevated in CP muscle relative to normal controls [8] suggesting that laminin β2 expression may be dysregulated in CP.

Laminin β2 knockout mice have defective NMJs characterized by sparse active zones, synaptic vesicles that are evenly distributed throughout the nerve terminal rather than concentrated at active zones, Schwann cell intrusion into the primary synaptic cleft, a decrease in the number of junctional folds, and impaired neuromuscular transmission [52], [55]. Laminin β2 knockout mice appear to have normally distributed AChE, however [28], suggesting that laminin β2 does not directly influence the distribution of AChE. It is, therefore, possible that pathways involved with laminin β2 and AChE are both dysregulated or that dysregulation of AChE is at the root of the NMJ disruption. Although qPCR data suggest similar levels of expression in CP and control groups (e.g., see Figure 3),details of AChE production, its association with ColQ and perlecan, and its stable localization in the NMJs of CP patients remain unclear and additional studies would be needed to clarify AChE localization in these patients.

Since both AChE and laminin β2 are associated with the synaptic basal lamina, our observations suggest that CP patients have inappropriately distributed basal lamina components in their NMJs. A recent report demonstrated that a patient with two truncating mutations in the gene encoding laminin β2 had a severe form of congenital myasthenic syndrome (CMS) characterized by severe muscle weakness [56]. An anconeous muscle biopsy from the patient revealed a predominance of type I fibers and a profound disruption in NMJ structure, including: small nerve terminals and small areas of apposition between the nerve and muscle, widened primary synaptic cleft, Schwann cell invasion of the synaptic space, reduction in the density of synaptic vesicles, and simplification of the postsynaptic membranes. Despite intact expression of AChE, the biopsy demonstrated reduced quantal content of endplate potentials (EPPs), providing a link between structural disruption of the basal lamina and NMJ function [56]. Similarly, several patients have been identified with endplate AChE deficiency (EAD), a CMS caused by mutations in the ColQ tail [57]. These mutations either prevent the binding of ColQ to AChE or the insertion of ColQ into the basal lamina, resulting in an absence of endplate AChE, prolonged endplate current, reduced quantal release by nerve impulse, and small nerve terminals that are often covered by Schwann cells [57]. Given that the structure of mature NMJs is tightly controlled and linked to neuro-muscle trophic interactions, we suspect that the mis-apposition of NMJ components in CP may be associated with altered NMJ molecular signaling and function.

Whether microanatomic differences in CP contribute to spasticity or the markedly reduced voluntary isometric force generation capacity observed in CP muscles [58], [59] requires further investigation. Nonetheless, the specific non-apposition between laminin β2 and AChE suggests that the basal lamina may be a key element in NMJ disorganization in CP muscles. Consistent with this finding, previous studies have shown that spastic muscle contains poorly organized ECM material compared to normal muscle [60] and altered gene expression patterns for critical proteins (including laminin β2) are found in CP muscle [8]. A recent study also demonstrated an increase in ECM material, including laminin and collagen, in muscle from children with CP [33]. Unfortunately, the mechanisms responsible for laminin distribution patterns within the muscle ECM are only partially understood, but the concept that ECM malformation is related to NMJ disorganization may warrant additional consideration.

The relationship between our current study and two previous studies is of particular note. Since the pharmacologic sensitivity to neuromotor blocking agents is linked to AChR, previous studies focused on possible expansion of the AChR distribution. In one prior study [9], two blinded investigators assessed images of NMJs taken from erector spinae muscle and stained for AChR and AChE; when the two agreed that a patient's sample included at least one NMJ with AChR extending beyond the limits of AChE, that patient was scored positive for non-junctional AChR. By that method, 28% of CP cases were identified as positive for disrupted NMJs versus 0% from the IS/control group. In the present study we also used erector spinae but a much more stringent and objective method was employed, and 16% of CP patients were identified as having significantly elevated AChR outside of AChE (i.e., ROE in Table 3; p<0.002). On average, however, AChR outside of AChE values for the erector spinae samples in the present study were not significantly different from the controls owing to the relatively low proportion of NMJs with elevated AChR outside of AChE among the total number of NMJs assessed. Interestingly, in a second prior study in which leg muscle from CP patients was assessed [13], a relatively high proportion of NMJs with elevated AChR outside of AChE were identified. In that study, a significant relationship was found between gross motor function and both the average AChR outside of AChE score and the proportion of highly dysmorphic NMJs present. Thus, among CP patients, the average AChR outside of AChE appears significantly higher in leg muscle than in back muscle, which may not be surprising given that the level of affect in CP tends to be clinically more severe in distal muscles. Overall, the present study and the previous two have a consistent finding that the muscle of CP patients contains dysmorphic NMJs.

### Neuromotor Maturational Status

In evaluating how the population of NMJs in individual CP patients differed from those of the IS control group, it was found that 84% of the CP patients had at least one localization mismatch that was significantly different from control. Consistent with the results determined when median values for each child were compared, AChE outside of laminin β2 was the most common mismatch. Beyond AChE outside of laminin β2, however, discernible patterns in the disruption of NMJs were not apparent. This lack of a general pattern in NMJ disruption may be due to patient-to-patient variation in responses to nerve-muscle dysregulation or related to the delay between the CNS injury that precipitated the disease and the evaluation of NMJs in the study. In either case, overall patterns associated with NMJ deformation in different children would be difficult to identify.

It should be noted that non-apposition of NMJ components like that evident in CP samples has been associated with a dynamic phase of muscle regeneration [61]. To assess whether the non-apposition seen in patients with CP was associated with cycles of regeneration, telomere lengths were examined. Telomeres are shortened in conditions where numerous cycles of muscle degeneration/regeneration take place like in DMD [62]. A lack of difference in telomere lengths between the IS and CP groups suggests that cycles of degeneration/regeneration were similar in the two groups and that recurrent muscle regeneration did not account for the observed lack of NMJ organization in CP versus IS muscles.

In our investigation of the maturational status of paraspinal muscle from CP and control patients, we found that the two groups appeared to have attained similar levels of neuromotor maturation. Histochemical analysis revealed that samples from CP and IS patients had the appearance of mature muscle. We did note a preponderance of slow twitch fibers in CP, which is in agreement with previous studies looking at muscle fiber types in CP and may result from differences in the mechanical work of the *spinalis* [36], [63]. It is not clear, however, why the muscle from the group of scoliotic patients with CP might differ from those with idiopathic disease in this regard.

The α1 and δ subunits of the AChR are thought to be expressed at all developmental stages in mammalian muscle; whereas, the ε subunit is expressed post-natally when it replaces the fetal γ subunit, which begins being expressed prior to innervation [11]. The α7 AChR subunit can also be expressed during development and denervation [41], [42], [64]. Analysis of these AChR subunits failed to reveal any significant differences in expression between CP and control groups. Similar results were found for myogenin, and the general results for the gene expression analysis support the notion that CP and IS muscles had both achieved the same degree of maturation with no evidence for denervation. Given these results, the up-regulation of β1-syntrophin in CP samples seems surprising as β1-syntrophin expression in myofibers generally correlates with AChRγ expression [65]. In our study, AChRγ was not altered in the CP patients, and the elevation of β1-syntrophin may be better accounted for by contributions from non-muscle tissues, like blood vessels and nerves [15] and other cell types [66] rather than a direct difference in myofibers.

To further assess NMJ maturation in CP and IS patients, we performed an additional phase of studies utilizing transmission electron microscopy (TEM) to examine the number and length of postsynaptic folds, which are critical indicators of NMJ maturation. Since localizing NMJs in muscle biopsies is a tedious and time consuming process, we devised a technique for rapid localization of NMJs using fluorescent bungarotoxin and tile mapping with confocal microscopy to find NMJs prior to final processing and visualization by electron microscopy [25]. The technique allows for rapid and accurate processing of human biopsy samples for TEM while minimizing structural changes that might occur with delayed muscle processing post-acquisition.

The general appearance of NMJs in CP samples was similar to that in controls. There was close apposition of nerve and muscle, distinct postsynaptic folding, and the absence of polyneuronal innervation, which all indicate that the two groups had attained a similar level of maturation. There were, however, some clear differences. Specifically, the NMJs from children with CP appeared to have fewer and longer synaptic folds compared to the IS patients and to previously published images of NMJs from human back muscle (Figure 4) [67]. Since the primary folds are rich in Na^+^ channels, which amplify the acetylcholine-induced signals transduced by AChRs, a functional consequence of increased fold length could be an alteration in the efficiency or duration of neurotransmission. For example, an increase in the extent of folding would be expected to decrease the threshold for the initiation of action potentials in the muscle fiber [67]. Thus, an increase in synaptic fold length and complexity may reflect functional differences in neuromotor transmission in the two groups; this idea may warrant further investigation.

Our observations also suggested that CP NMJs have a decreased mitochondrial load in the nerve terminal as compared to controls. We corroborated our ultrastructural findings by immunoflourescence staining of mitochondria. Mitochondrial accumulation at the nerve terminal is mediated through the actin cytoskeleton, and high levels of mitochondria are needed to support neurotransmitter release and nerve terminal function [45], [46]. Studies indicate that presynaptic mitochondria perform additional functions besides providing the energy for synaptic release of vesicles and they may participate in synaptic vesicle biogenesis by driving ATP-dependent vacuolar proton pumps in the synaptic vesicle membrane, which act in the refilling of neurotransmitters into its lumen [68]. It has also been suggested that calcium ions sequestered in mitochondria can provide a source for the potentiation of neurotransmitter release [69]. The ATP produced by mitochondria is also co-packaged into synaptic vesicles and co-released with acetylcholine. The released ATP acts as a neurotransmitter stimulating purinergic receptors that play key roles in the development and maturation of the NMJ [70]. A lack of mitochondria would be expected to alter purinergic metabolism at the NMJs of CP patients, and based on the strong association between synaptic structure and the normally high “safety factor” of mammalian NMJs, our results suggest that the efficiency of neurotransmission to muscles in CP may be compromised.

The ultrastructural differences observed in the spastic CP NMJs are similar to those described in certain genetic diseases and in knockout mice that lack key basal lamina components. At human NMJs, the pattern of postsynaptic membrane folding can be complex; however, the depth of synaptic folds is very consistent [71]. A few neuromuscular disorders such as Bethlem myopathy [72] and limb-girdle myasthenia syndrome [67] have been identified with ultrastucturally disorganized NMJs. In particular, immunoblotting studies of NMJs from patients with limb girdle myasthenia showed essentially normal levels of 14 different NMJ proteins, including components of the basal lamina, postsynaptic membrane, and postsynaptic cytoskeleton; however, on ultrastructural examination, the depth of postsynaptic folding was found to be only half that of controls [67]. In knockouts of the laminin β2 subunit, which is present in all three forms of synaptic laminin trimers found in muscle (i.e., laminins 4, 9, and 11) and has been linked to pre-terminal differentiation [30], mice had fewer post-junctional folds than wild type mice [73]. In addition, mice lacking the laminin α4 subunit (found in laminin 9) have presynaptic active zones and junctional folds in normal numbers, but these are not well apposed to each other [72]. The role of laminin α5, which is present in the laminin 11 trimer, has been difficult to assess *in vivo* because α5 deficiency is embryonically lethal due to extra-muscular defects [74]; however, *in vitro* assays indicate that α5 inhibits motor axon growth and prevents Schwann cells from entering the synaptic cleft [51], [75], [76]. Thus, synaptic laminins are critical determinant of NMJ conformation. In addition, proteins of the DGC complex, which links the BL to the intracellular actin cytoskeleton, also play important roles in the structural integrity of the NMJ. For example, utrophin is normally localized with AChRs at the crest of the postsynaptic junctional folds [77], [78], and utrophin mutant mice exhibit a lower density of AChRs at the NMJs and fewer junctional fold openings [79], [80]. Although further work is needed to test the idea, the ultrastructural defects present in the NMJs of children with spastic CP may be related to alterations in basal lamina proteins like laminin or BL-associated DGC components like utrophin.

Our observation of patterns in the structural and developmental measures assessed in this study suggests that differences in maturational status do not account for dysmorphism in the NMJs of children with spastic CP. Alternative explanations for the observed differences seen in CP versus control samples include inappropriate trophic interactions at the NMJ due to altered molecular signaling from lower motor neurons during recovery from a CNS injury suffered during neuromotor maturation. Other alternatives include the possibility that inappropriately organized NMJs may develop as an adaptive response to the years of altered activity, weakness, poor coordination, and spasticity that CP patients have accumulated or that the disorganization may occur as a primary result of the incident or infection that caused the CNS injury. One compelling possibility is that specific windows of developmental opportunity may exist during neuromotor maturation and that one or some of these are missed when a CNS injury interrupts normal NMJ development. Such alternative explanations for NMJ deformations in CP require further research to evaluate; however, linking peripheral nervous system deficits with CP and elucidating the pathways leading to peripheral disruption and disordered movement may provide targets for advanced therapeutic intervention to treat motor disability in CP.

## Supporting Information

Figure S1 **Laminin β2 2 distribution in** ***Spinalis*** **.** A *Spinalis* sample from an IS patient was double stained with C4 anti- laminin β2 antibody (green) and tetramethylrhodamine-conjugated bungarotoxin (Btx). Although laminin β2 expression was evident along the sarcolemma in human samples, the thresholding algorithm could delimit the NMJ by the more intense fluorescence signal.
(TIF)

Figure S2 **Colocalization of AE-2 anti-AChE staining and fasciculin-2.** A *Spinalis* sample from an IS patient was double stained with purified AE-2 anti-AChE antibody (green) and Alexa Fluor 594-labeled fasciculin-2, a purified snake toxin that binds AChE with high specificity and high affinity. The two stains colocalized, indicating that both specifically label AChE.
(TIF)

Text S1 **Detailed Methods. Table S1, Patient demographics. Table S2, Primer sets used for Real Time PCR. Table S3, Inter-rater reliability for TEM Image Analyses. Table S4, Antibody Probes tested.**
(DOCX)
