# Supplementary figures and images for: Disruption of Basal Lamina Components in Neuromotor Synapses of Children with Spastic Quadriplegic Cerebral Palsy
Source: PLoS One. 2013 Aug 16;8(8):e70288. doi: 10.1371/journal.pone.0070288 (PMC3745387; doi:10.1371/journal.pone.0070288)

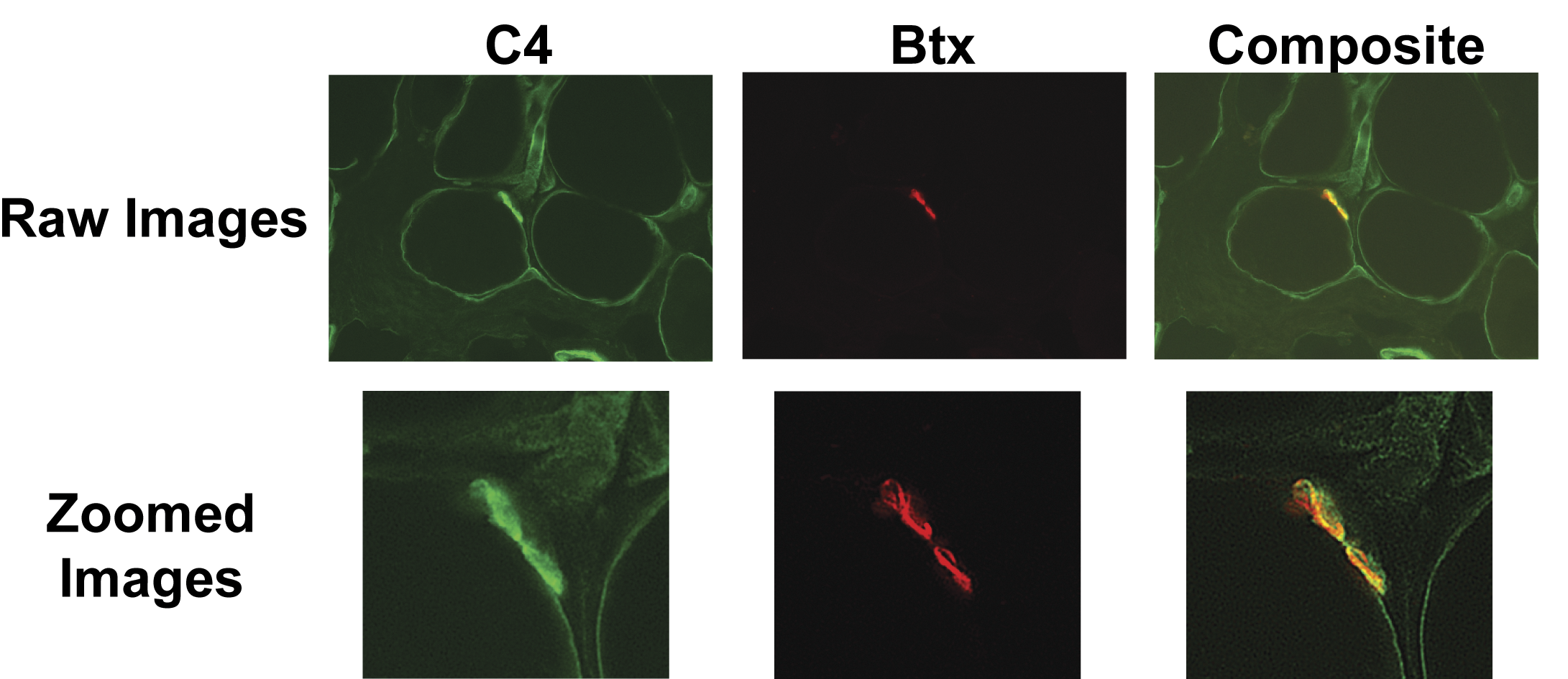

Supplement: Figure S1 — Laminin β2 2 distribution in Spinalis . A Spinalis sample from an IS patient was double stained with C4 anti- laminin β2 antibody (green) and tetramethylrhodamine-conjugated bungarotoxin (Btx). Although laminin β2 expression was evident along the sarcolemma in human samples, the thresholding algorithm could delimit the NMJ by the more intense fluorescence signal. (TIF) [file pone.0070288.s001.tif]

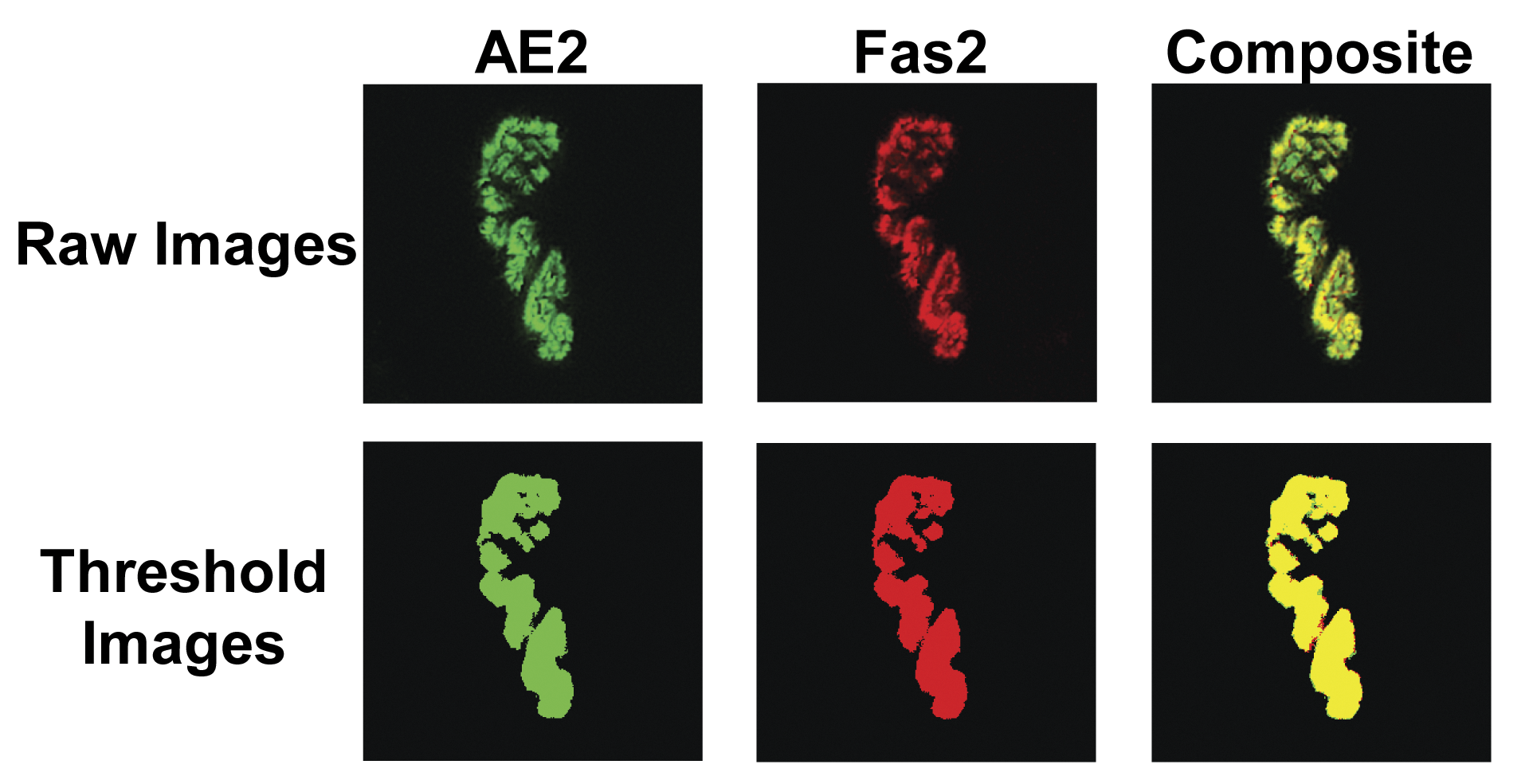

Supplement: Figure S2 — Colocalization of AE-2 anti-AChE staining and fasciculin-2. A Spinalis sample from an IS patient was double stained with purified AE-2 anti-AChE antibody (green) and Alexa Fluor 594-labeled fasciculin-2, a purified snake toxin that binds AChE with high specificity and high affinity. The two stains colocalized, indicating that both specifically label AChE. (TIF) [file pone.0070288.s002.tif]
