# Supplementary material for: Disruption of Basal Lamina Components in Neuromotor Synapses of Children with Spastic Quadriplegic Cerebral Palsy
Source: PLoS One. 2013 Aug 16;8(8):e70288. doi: 10.1371/journal.pone.0070288 (PMC3745387; doi:10.1371/journal.pone.0070288)
Supplement: Text S1 — Detailed Methods. Table S1, Patient demographics. Table S2, Primer sets used for Real Time PCR. Table S3, Inter-rater reliability for TEM Image Analyses. Table S4, Antibody Probes tested. (DOCX) [file pone.0070288.s003.docx]

# Supporting Information

## Detailed Methods

*Patient selection and exclusionary criteria*. Patients carrying a diagnosis of neuromuscular scoliosis other than CP or a diagnosis of any chromosomal disorder, degenerative neurological disease, dystrophy, or dystroglycanopathy were excluded. Patients who had received any injections of botulinum toxin to relieve spasticity within 12 months prior to surgery were also excluded.

*Fluorescence staining*. Slides containing 8 μm thick cryosections of samples of spinalis were fixed at room temperature for 5 minutes using 10% neutral buffered formalin (NBF; Fisher) and blocked at room temperature for 30 minutes using 3% BSA (Sigma) in PBS. The supernatant collected from AE-2 hybridoma cells (ATCC), was purified using a Protein G column (KPL). The purified antibody was concentrated, desalted, and brought up in Dulbecco’s phosphate buffered saline (D-PBS) using Microcon YM-30 centrifugal filters (Millipore). The concentration of the antibody was determined using a BCA assay (Pierce) and the antibody was biotinylated using a FluoReporter Mini-Biotin-XX Protein Labeling Kit (Molecular Probes). The free biotin was removed using a spin column provided with the kit, followed by centrifugation through a Microcon YM-30 filter. Sections were stained in a solution containing 0.16 μg/mL tetramethylrhodamine-conjugated α-bungarotoxin (Btx; Molecular Probes) for 1 h at room temperature. The sections were then stained using hybridoma supernatant containing either a monoclonal antibody to laminin β2 (C4; Developmental Studies Hybridoma Bank [DSHB]; Figure S1), diluted 1:10, or hybridoma supernatant containing a monoclonal antibody to SV2 (DSHB) for 1 h, followed by 10 μg/mL Alexa Fluor 488-conjugated secondary antibody (Molecular Probes) for 1 h. Subsequently, sections were incubated with 120 μg/mL unconjugated mouse IgG (Sigma) for 30 minutes to block any unbound secondary antibody, then incubated with 3 μg/mL biotinylated AE-2 antibody for 1 h, followed by 10 μg/mL Alexa Fluor 647-conjugated streptavidin (Molecular Probes) for 1 h. The sections were rinsed extensively with D-PBS between steps.

Our selection of the AE-2 antibody was based on four considerations: (i) its specificity for AChE as detailed in the literature,[1] (ii) its high signal to noise ratio in staining normal human NMJs in our hands, (iii) the co-localization of its staining pattern with fluorescently-tagged fasciculin-2, a purified snake toxin that binds AChE with high specificity and high affinity (Kd =25 pM),[2,3,4] and (iv) its relatively low cost given the number of sections being evaluated.

For comparison of AE-2 and fasciculin-2 (FAS2) staining patterns, FAS2 (Alomone Labs) was labeled using an Alexa Fluor 594 Microscale Protein Labeling Kit (Molecular Probes). Free dye was removed using spin columns containing Bio-Gel P2 Gel (BioRad), and the final protein concentration and degree of labeling were determined using a NanoDrop ND-1000. Samples were stained using the Alexa Fluor 594-conjugated FAS2 at 0.6 μg/mL and purified AE-2 with an Alexa Fluor 488-conjugated secondary antibody (Figure S2).

*Calculation of appositional score*. Briefly, two-color composite images were formed by merging images from the same section and determining the boundaries of each staining pattern using a thresholding algorithm. One stain pattern was pseudo-colored red and the other green so that areas of overlap appeared yellow and background appeared black. The degree of colocalization was determined by calculating both the fraction of red pixels exhibiting red stain but no green [Red/(Red + Yellow)] and the fraction of green pixels exhibiting green stain but no red [Green/(Green + Yellow)]. These calculations resulted in six values for each NMJ: AChR outside AChE, AChE outside AChR, target molecule (SV2 or laminin β2) outside AChE, AChE outside target molecule, target molecule outside AChR, and AChR outside target molecule. These values are dimensionless variables indicating proportional areas and the degree of functional apposition.*Telomere length analysis*. Genomic DNA (n = 10 patients with CP, 10 patients with idiopathic scoliosis, 3 patients with Duchenne’s Muscular Dystrophy) was extracted using an ArchivePure DNA Cell/Tissue Kit (5PRIME), concentration was determined on a NanoDrop, and purified DNA was diluted to 1.75 ng/μL in TE. Telomere (T) PCRs and single copy gene (S) PCRs were performed in separate 96-well plates using previously published primer sequences [5]. PCR reactions were performed in a total volume of 50 μL, containing 35 ng of DNA, 1x iQ SYBR Green Supermix, 270 nM of telomere 1 primer and 900 nM of telomere 2 primer, or 300 nM of 36B4u primer and 500 nM of 36B4d primer. The real-time PCR was performed on a MiIQ thermocycler with the following amplification protocol: 95°C for 10 min, followed by 40 cycles of 95°C for 15 s and 54°C for 2 min. Each reaction was run in triplicate and a standard curve was included containing reference DNA diluted serially 1.68-fold to produce concentrations of DNA ranging from 0.63 – 5 ng/μL. The efficiency of each primer set was determined from the standard curves (93% for the telomere primers, 100% for the 36B4 primers).

*Sample Preparation for Electron Microscopy and Analysis of Mitochondrial Content.* Samples were fixed overnight with 4% paraformaldehyde (PFA) in 0.1M Sorensen’s phosphate buffer at pH 7.4 (PB), and rinsed three times in PB followed by infiltration with 2.3 M sucrose (Fisher) in PB using a series of dilutions (25%, 33%, 50%, 67%, 75%, 100%, and 100%) for one hour each at room temperature followed by storage at 4oC in 2.3 M sucrose overnight. Tissue was embedded in Tissue Freezing Medium (EMS) and cryosectioned at 10 µm thickness using a Leica CM3050-S cryostat and placed on 4X adhesive-coated slides (EMS). Slides were blocked with 3% BSA (Sigma) in PB and stained with Alexa Fluor 555-conjugated α-bungarotoxin (Invitrogen). NMJs were located and tile mapped as previously described in detail.[6] Briefly, sections were imaged by fluorescence microscopy to locate NMJs based on an X-Y coordinate grid. The tile-mapped slides were re-fixed overnight at 4°C in 2% glutaraldehyde with 2% PFA (EMS) in 0.1 M sodium cacodylate buffer, pH 7.4 (EMS). The sections were rinsed 3x, 15 min each, in 0.1 M sodium cacodylate buffer and post-fixed for 2 h in 1% osmium tetroxide (EMS, Hatfield, PA) in 0.1M cacodylate buffer. The slides were washed 2x in cacodylate buffer and 2x in water then dehydrated in a graded ethanol (EMS; 25%, 50%, 75%, 95%, 100%, 100% for 15 min each) and infiltrated with dilutions of Embed-812 resin (EMS) in ethanol (25%, 33%, 50%, 67%, 75%, 100% and 100% for 1 h each) followed by overnight infiltration in fresh 100% resin. The sections were inverted onto resin-filled slide-duplicating molds (Ted Pella, Inc.), and polymerized at 60°C for 48 h. Regions of interest were identified based on the tile map coordinates and isolated with a diamond scribe. Samples were mounted onto pre- polymerized, resin-filled BEEM capsules using cyanoacrylate glue and trimmed to encompass multiple NMJs referring again to the tile coordinates for reference. Alternating sets of ultrathin (60-70 nm) and semi-thin (200 nm) sections were collected with a Reichert-Jung Ultracut E ultramicrotome. Semi-thin sections were dried onto glass slides, stained with epoxy tissue stain (EMS), and imaged with a Zeiss Axioskop 2 equipped with a Zeiss Axiocam. The semi-thin sections in conjunction with the image of the resin block face served as references to identify mapped muscle fibers. Ultrathin sections were collected onto 200 mesh Formvar-carbon coated copper grids stabilized with evaporated carbon film (EMS), stained with methanolic uranyl acetate and Reynolds’ lead citrate,[7] and viewed with a Zeiss Libra 120 Transmission Electron Microscope at 120kV. Images were captured with a Gatan Ultrascan 1000 digital camera.

For mitochondrial analysis, all samples were processed and imaged in parallel to minimize variability. Similar to the approach for TEM, muscle samples were collected in 4% PFA in 0.1M PB, cut into 1 mm x 2 mm pieces, and fixed overnight. The tissue was rinsed in 0.1 M PB and infiltrated with 2.3 M sucrose using as for the TEM samples. Multiple bundles of cryo-protected muscle were oriented longitudinally in a disposable base mold (EMS, Hatfield, PA), excess sucrose was removed with filter paper, and the muscle was embedded in Tissue Freezing Medium ^TM^ (EMS, Hatfield, PA) and frozen in a Leica CM3050 S cryostat. Ten micron thick cryosections were collected onto cold adhesive microscope slides (EMS, Hatfield, PA) using the CryoJane® Tape-Transfer System (Instrumedics, Inc., St Louis MO).

*Statistical Analysis*. Statistical analyses were carried out using SPSS 11.5 software. Non-parametric Mann-Whitney tests were used to compare median appositional scores between the CP and idiopathic scoliosis populations. Additionally, median values were determined for each child and compared between the two groups. Pairwise Mann-Whitney tests were used with a Bonferroni correction to compare individual CP patients with the values determined for the control group. Binary logistic regression was used to assess which appositional scores were most predictive of a diagnosis of CP versus idiopathic scoliosis. A Kruskal-Wallis test was used to compare T/S ratios. In all cases, significance was assessed at p < 0.05 unless otherwise noted.

## Results

*Patient demographics*. Data regarding height and angle of spine curvature were also collected from both groups (**Table S1**). Among these, weight, height, gender, and angle of spine curvature were each found to differ between the two groups. In subsequent analyses, no correlations were found between the degree of NMJ component mis-apposition (see below) and the age, weight, height, gender, or angle of spine curvature associated with patients within the CP population (Spearman’s rho << 0.3 for all comparisons).

*The Distribution of NMJ Components is disrupted in Children with Spastic CP.* The mean ROE for *spinalis* from patients with CP (0.07 ± 0.09, n = 1589 NMJs total) was substantially different from the values determined in a previous study of leg muscles (gracilus, vastus lateralis, and gastrocnemius) from 59 CP patients (0.16 ± 0.08, n = 853 NMJs for ambulatory patients and 0.23 ± 0.14, n = 2060 NMJs for non-ambulatory patients).[8] To determine if the type of muscle analyzed contributed to ROE values, an analysis was performed using the *spinalis* data of three CP patients who happened to be enrolled in both studies and comparing it to the previously acquired data. For each patient, median ROE values were consistently higher in leg muscles than in *spinalis*: Patient A, ROE_leg_=0.09 vs. ROE_back_=0.04; Patient B, ROE_leg_=0.16 vs. ROE_back_=0.03; Patient C, and ROE_leg_=0.24 vs. ROE_back_=0.05. The degree of disruption may be significantly higher in leg muscle versus back muscle; however, back muscle was used in the present study because of the availability of control/non-CP samples from the same anatomic location.

## Discussion

When the ROE values for the back and leg muscles were compared in the same patients, ROE was found to be consistently higher in the leg muscles. This suggests that leg muscles contain more severely affected NMJs than *spinalis* in children with CP, a result that is consistent with the clinical presentation of these children. Given that the NMJs in back muscles are clinically less affected in CP than leg muscles, it seems likely that the differences found in the current study represent more severe structural and functional abnormalities than the previously reported mismatches between AChR and AChE.[8,9]

**References**

1. Fambrough DM, Engel AG, Rosenberry TL (1982) Acetylcholinesterase of human erythrocytes and neuromuscular junctions: homologies revealed by monoclonal antibodies. Proc Natl Acad Sci U S A 79: 1078-1082.

2. Anglister L, Eichler J, Szabo M, Haesaert B, Salpeter MM (1998) 125I-labeled fasciculin 2: a new tool for quantitation of acetylcholinesterase densities at synaptic sites by EM-autoradiography. J Neurosci Methods 81: 63-71.

3. Marchot P, Khelif A, Ji YH, Mansuelle P, Bougis PE (1993) Binding of 125I-fasciculin to rat brain acetylcholinesterase. The complex still binds diisopropyl fluorophosphate. J Biol Chem 268: 12458-12467.

4. Rotundo RL (2003) Expression and localization of acetylcholinesterase at the neuromuscular junction. J Neurocytol 32: 743-766.

5. Cawthon RM (2002) Telomere measurement by quantitative PCR. Nucleic Acids Res 30: e47.

6. Modla S, Mendonca J, Czymmek KJ, Akins RE (2010) Identification of neuromuscular junctions by correlative confocal and transmission electron microscopy. J Neurosci Methods 191: 158-165.

7. Reynolds ES (1963) The use of lead citrate at high pH as an electron-opaque stain in electron microscopy. J Cell Biol 17: 208-212.

8. Theroux MC, Oberman KG, Lahaye J, Boyce BA, Duhadaway D, et al. (2005) Dysmorphic neuromuscular junctions associated with motor ability in cerebral palsy. Muscle Nerve 32: 626-632.

9. Theroux MC, Akins RE, Barone C, Boyce B, Miller F, et al. (2002) Neuromuscular junctions in cerebral palsy: presence of extrajunctional acetylcholine receptors. Anesthesiology 96: 330-335.

## Supporting Tables and Figure

Table S1: Patient demographics.

| **Parameter** | **Idiopathic scoliosis** | **Cerebral palsy** |
| --- | --- | --- |
| **Age (years)** | 13.9 ± 1.9 (36) | 13.0 ± 3.1 (34) |
| **Weight (kg) ^*^** | 60.2 ± 16.1 (36) | 32.2 ± 8.2 (33) |
| **Height (cm) ^*^** | 162.6 ± 10.4 (36) | 136.6 ± 12.2 (29) |
| **Gender distribution *** | 30 female, 6 male | 20 female, 14 male |
| **Angle of spine curvature *** | 50.5 ± 9.9 (36) | 66.3 ± 19.2 (34) |

Data presented as actual or as mean ± S.D. (n) for all patients included in the studies. Asterisks indicate parameters showing a difference between the two groups that is statistically significant (p < 0.05 by Chi Square, for gender, or Mann-Whitney for all others). Note that data regarding height and weight were missing from the charts of some CP patients. Weights measured with a brace were excluded.

Table S2: Primer sets used for Real Time PCR.

| **Gene Name** | **Primer Sequence** |
| --- | --- |
| β-Glucuronidase | F: 5' CTCATTTGGAATTTTGCCGATT 3'  R: 5' CCGAGTGAAGATCCCCTTTTTA 3' |
| Cystic Fibrosis Receptor | F: 5' TAGGAAGTCACCAAAGCAGTACAGC 3'  R: 5' AGCTATTCTCATCTGCATTCCAATG 3' |
| 18s Ribosomal RNA (18s) | F: 5' CTTAGAGGGACAAGTGGCG 3'  R: 5' ACGCTGAGCCAGTCAGTGTA 3' |
| Alpha Tubulin | F: 5' CATTGATGAAGTTCGCACTGG 3'  R: 5' GATCTCCTTGCCAATGGTGTA 3' |
| β-2-Microglobulin | F: 5' GCAAGGACTGGTCTTTCTATCTCTTG 3'  R: 5' TCAACCTCCATGATGCTGCTTAC 3' |
| Acetylcholine Receptor α | F: 5' CCGTCTGGTGGCAAAGCTATTTAA 3'  R: 5' GGTTGTAATCCACCCATTGCTGT 3' |
| Acetylcholine Receptor δ | F: 5' CCCTCACACTCTCCAACCTCAT 3'  R: 5' CCAGCACAATCTCTGGGAGCCA 3' |
| Acetylcholine Receptor γ | F: 5' GCTCGCAGACCTGATGCAAA 3'  R: 5' CGCACCACTGCATCTCTATCCA 3' |
| Acetylcholine Receptor ε | F: 5' GGATTGGAATCGATTGGCAGGATT 3'  R: 5' CCGAACTGGCCATCAATATTGTT 3' |
| β1 Syntrophin | F: 5' GCACTGCTTGCACCTACAAAA 3'  R: 5' GGTCCAGTTGAATCTCTCCATCTT 3' |
| β2 Syntrophin | F: 5' GCTGATCAAGGAAGTCTCTCTA 3'  R: 5' AAGAGTGCAGGTCCATGGTCAGTT 3' |
| Myogenin | F: 5' GCGCAGTGCCATCCAGTACAT 3'  R: 5' CGTGAGCAGATGATCCCCTGGGTT 3' |
| Acetylcholine Esterase | F: 5’GGCTACGAGATCGAGTTCATCT 3’  R: 5’GTGTCGGTGGCGCTGAGCAATTT 3’ |

Table S3: Inter-rater reliability for TEM Image Analyses

| **Measure** | **Method** | **Measures in 10 samples** | **Comparison** | **Statistic** |
| --- | --- | --- | --- | --- |
| Depth of primary fold | Trace fold from origin at the cleft to the furthest point that is continuous and unbroken. | 119 | Pearson Correlation | R2 = 0.993 |
| Distance between primary folds | Measure distance between the closest edges of two neighboring folds | 106 | Pearson Correlation | R2 = 0.997 |
| Cross- sectional area of mitochondria | Demarcate each mitochondrion with in the nerve terminal and calculate the area within divided by the area without | 73 | Pearson Correlation | R2 = 0.995 |

Table S4: Antibody Probes tested.

| **NMJ Target** | **Source** | **Catalog Number** | **Dilutions Tested** | **Readily Detected Over Background?** |
| --- | --- | --- | --- | --- |
| AChE | DSHB | AE-2 | 1:50 (purified, biotinylated) | Yes |
| Bassoon | Abcam | ab110426 | 1:50, 1:100, 1:500 | No |
| Bassoon | Novus | NBP1-80595 | 1:50, 1:100, 1:500 | No |
| ErbB4 | Oncogene | OP122 | 1:50, 1:100 | No |
| Laminin β2 | DSHB | C4 | 1:10 (supernatant) | Yes |
| MuSK | Abgent | AP7664a | 1:20, 1:50, 1:100 | Very faint |
| Neuregulin | Lab Vision | MS-272-P0 | 1:50, 1:100 | Very faint |
| NSF | BD Transduction Labs | 612272 | 1:10, 1:20, 1:50, 1:100 | Very faint |
| Piccolo | Abcam | ab110427 | 1:200, 1:500 | No |
| SNAP-25 | Stressgen | VAM-SV012 | 1:10 | No |
| SV2 | DSHB | SV2 | Neat (supernatant) | Yes |
| Syntaxin 1A | Abcam | ab41453 | 1:100, 1:500, 1:2000 | Very faint |
| Syntaxin 1B | Synaptic Systems | 110402 | 1:100, 1:1000 | Very faint |
| Syntrophin | Affinity BioReagents | MA1-745 | 1:10 | Non-specific |
| VAMP | Chemicon | MAB335 | 1:100, 1:200 | No |
| VAMP2 | Stressgen | VAS-SV006 | 1:50, 1:100 | Non-specific |
